# Implementation of Genetic Testing in Prostate Cancer: A Real-World Survey of Outpatient Urologists in Germany (PRO-GEN)

**DOI:** 10.3390/cancers18132030

**Published:** 2026-06-23

**Authors:** Julia C. Kaulfuss, Jonathan Jeutner, Barbara Erber, Carolin Siech, Mike Wenzel, Felix K. H. Chun, Eva Hellmis, Christian P. Meyer, Thorsten Schlomm, Maria De Santis, Nadine Biernath

**Affiliations:** 1 Charité—Universitätsmedizin Berlin, Corporate Member of Freie Universität Berlin and Humboldt-Universität zu Berlin, Department of Urology, 10117 Berlin, Germany; 2 Goethe University Frankfurt, University Hospital, Department of Urology, 60596 Frankfurt am Main, Germany; 3 Urologicum Duisburg, 47169 Duisburg, Germany; 4 Ruhr-University Bochum, Herford Hospital, Department of Urology, 32049 Herford, Germany; 5 Medizinische Universität Wien, Department of Urology, 1090 Vienna, Austria

**Keywords:** genetic testing, germline testing, prostate cancer, multi-center survey

## Abstract

**Background/Objectives**: Genetic testing (GT) is essential for precision therapy in prostate cancer (PC). Implementation in routine outpatient cancer care remains heterogeneous. We assessed real-world data on GT practices among German Outpatient Urologists (GOUs) to identify strategies for improvement. **Methods**: A nationwide, multi-center survey was conducted among GOUs in Germany (02–06/2025), assessing demographics, qualifications, and GT behavior. Cochran-Armitage test for trend and Fisher’s exact test were used to assess associations; odds ratios with 95% confidence intervals were estimated using binary logistic regression. **Results**: In total, 117 GOUs participated, all practiced in outpatient settings. GT differed by disease stage: in localized prostate cancer, 6.0% reported ordering somatic testing (SoT) and 8.5% germline testing (GeT), compared to 67.5% and 42.7%, respectively, in metastatic disease. Overall, 29.1% reported not ordering GT of any kind. Ordering SoT for metastatic disease was significantly lower among senior physicians compared to early- and mid-career physicians (43% vs. 78% and 74%; OR 0.46 per career stage, 95% CI 0.27–0.79, *p* = 0.004). GeT did not differ significantly by experience (OR 0.90 per career stage, 95% CI 0.56–1.46, *p* = 0.67). Neither gender (OR 0.74, 95% CI 0.31–1.74), nor having a qualification in urological cancer-specific genetic counseling (26.5% of respondents; OR 0.81, 95% CI 0.33–1.98) was significantly associated with testing. BRCA1 and BRCA2 were recognized by 96.6% of respondents. Knowledge of other PC-associated genes was much lower. **Conclusions**: Despite guideline recommendations, GT, particularly GeT, remains underutilized in PC care in Germany. Senior physicians tested significantly less, and gene knowledge beyond BRCA1/2 was limited.

## Simple Summary

Prostate cancer patients with genetic mutations can benefit from targeted therapies such as PARP inhibitors. Therefore, genetic testing is becoming increasingly important for treatment decisions and for identifying families with hereditary cancer risk. However, there is little real-world data about how genetic testing is implemented in routine prostate cancer care in Germany. In this nationwide survey, we assessed current knowledge and testing practices among German Outpatient Urologists who perform routine prostate cancer care. We found that genetic testing, particularly germline testing, is still underutilized despite international guideline recommendations and growing therapeutic relevance. Our findings highlight important gaps between guideline recommendations and real-world clinical practice and underline the need for a broader implementation of genetic testing and precision oncology in routine outpatient prostate cancer care.

## 1. Introduction

Prostate cancer is the most common oncological disease affecting men in Europe [1]. In addition to multifactorial etiological factors, genetic alterations play a significant role in the development and progression of prostate cancer. The importance of genetic testing (GT) in prostate cancer patients is continuously rising as about 5–15% of metastatic and localized prostate cancer cases present genetic mutations in homologous recombination repair (HRR) genes and mismatch repair (MMR) genes [2,3]. The characterization of a patient’s genetic profile not only informs familial risk but also provides important prognostic and therapeutic insights enabling precision oncology.

Current international guidelines recommend GT in prostate cancer, although there are some differences regarding clinical stage, risk, pretreatment, and family history. The NCCN guidelines adopt the most comprehensive approach, extending germline testing (GeT) to patients with high- and very-high-risk localized disease in addition to metastatic disease [4]. The EAU guidelines consider the family history, known hereditary syndromes, and therapeutic relevance as important factors rather than recommending GeT in all metastatic cases [5]. The German S3 guideline limits GeT in the localized setting mainly to patients with suspected hereditary cancer syndromes but clearly recommends genetic counseling and GeT for all men with metastatic prostate cancer. Particularly, HRR mutation testing prior to metastatic castration resistant prostate cancer (mCRPC) treatment is recommended [6]. Overall, while there is broad consensus on the importance of GT in advanced metastatic disease, the timing, indications, and extent of GT in localized disease differ between guidelines. Despite guideline recommendations and the rising clinical importance of GT, real-world data on the knowledge and implementation of GT in prostate cancer remain limited [2,7,8,9]. Throughout this manuscript, guideline-concordant practice was primarily assessed against the German S3 Guideline on PC, representing the national standard of care. The EAU Guidelines were additionally considered because they are widely used in Germany and, due to more frequent updates, include recent evidence and clinical developments earlier.

Our study aimed to collect and analyze real-world data on GT, and especially germline testing, practices in Germany by surveying GOUs, who provide the majority of prostate cancer care.

## 2. Materials and Methods

We conducted a nationwide multi-center online survey among German Outpatient Urologists (GOUs) across Germany. The active survey period lasted four months, from February to June 2025. The overall study process, including study conception, questionnaire development, ethical approval, data collection, and data analysis, was conducted between December 2024 and January 2026.

The survey was distributed nationwide in Germany via different channels:

In total, 438 GOUs were contacted directly by email and clinical newsletters from two university urology departments (Goethe University Frankfurt University Hospital, and Ruhr University Bochum, Herford Hospital), reaching approximately 210 and 25 outpatient urologists, respectively, and a brief study announcement including the survey link was published in UroForum, a German professional journal for urologists. In total, about 3605 urologists practice as GOUs in Germany. Because recipients may have been exposed through more than one distribution channel, and the number of readers who viewed the journal announcement was not available, the total number of unique urologists reached could not be determined. A total of 117 GOUs completed the survey. The online survey contained 18 multiple-choice and single-choice items. (Table 1) The questions addressed the GOU’s demographics, testing behavior, clinical situations triggering GT, family history, clinical referral pathways, and the use of interdisciplinary tumor boards. The survey questions addressed the two common ways to detect genetic alterations: SoT, defined as the analysis of acquired genetic alterations in tumor tissue or liquid biopsies, and GeT, defined as the analysis of inherited variants. These definitions were not displayed in the questionnaire, as knowledge of this distinction was considered essential for physicians involved in prostate cancer care. The survey was conducted electronically, and responses were collected anonymously. Statistical analysis was descriptive for demographic variables. The Cochran-Armitage test for trend was used to assess associations between testing behavior and years of clinical experience. The original seven response categories were predefined and reduced to three ordinal groups (early career 1–10 years, mid-career 11–20 years, and senior > 20 years) due to small cell sizes in the individual strata. McNemar’s exact test was used for paired comparisons of testing rates between disease stages. Fisher’s exact test was used to compare testing rates by gender and by genetic counseling qualification. A two-sided significance level of α = 0.05 was applied. Analyses were performed using Python 3.12 (scipy, statsmodels). The study and the questionnaire were reviewed and approved by the responsible institutional ethics committee (EA4/147/25).

## 3. Results

### 3.1. Demographics

**Figure 1:**
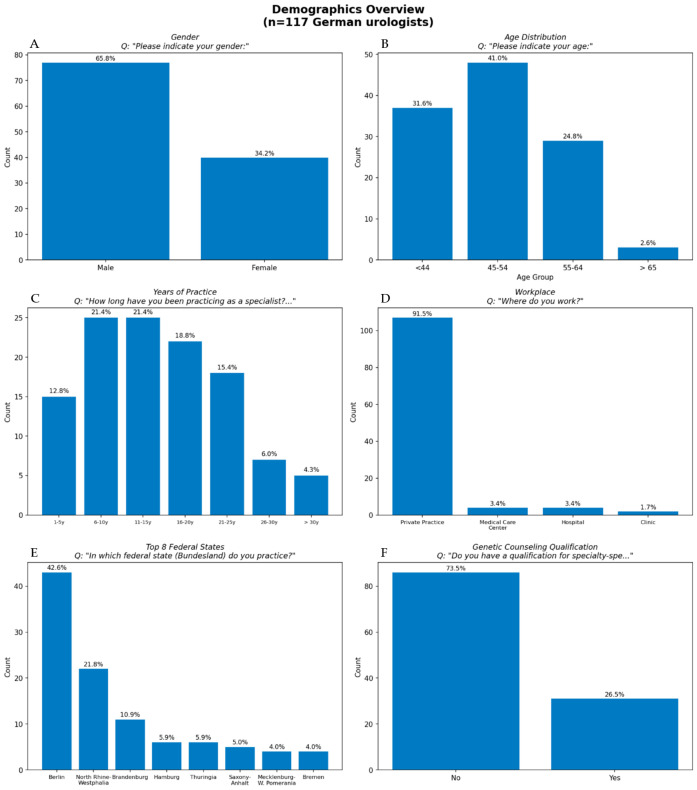
Demographics summary. Overview of respondent demographics (n = 117 German urologists). (**A**) Gender distribution (Q: “Please indicate your gender.”). (**B**) Age distribution (Q: “Please indicate your age.”). (**C**) Years of specialist practice (Q: “How long have you been practicing as a specialist?”). (**D**) Workplace setting (Q: “Where do you work?”). (**E**) Geographic distribution by the top 8 federal states (Q: “In which federal state do you practice?”). (**F**) Genetic counseling qualification (Q: “Do you have a qualification for specialty-specific genetic counseling?”). Responses are shown as bar charts with absolute counts; percentages are annotated where applicable.

A total of 117 GOUs participated in the survey. The total reach of journal and newsletter distribution cannot be quantified, but based on direct emails to GOUs alone, the minimum response rate was 27% (117/438). Of these, 65.8% were male and 34.2% female. The largest age group was 45–54 years (41.0%), followed by physicians younger than 44 years (31.6%) and the group aged 55–64 years (24.8%). Most respondents were working as GOUs in outpatient practices (office-based) (91.5%), with smaller proportions practicing as GOUs in outpatient medical care centers (3.4%). Regarding professional experience, the largest groups reported 6–10 years (21.4%) and 11–15 years (21.4%) of specialized practice in urology. Thirty-one (31/117, 26.5%) of all respondents reported having an additional qualification in urological cancer-specific genetic counseling (Figure 1).

### 3.2. Genetic Testing and Disease Stage

**Figure 2:**
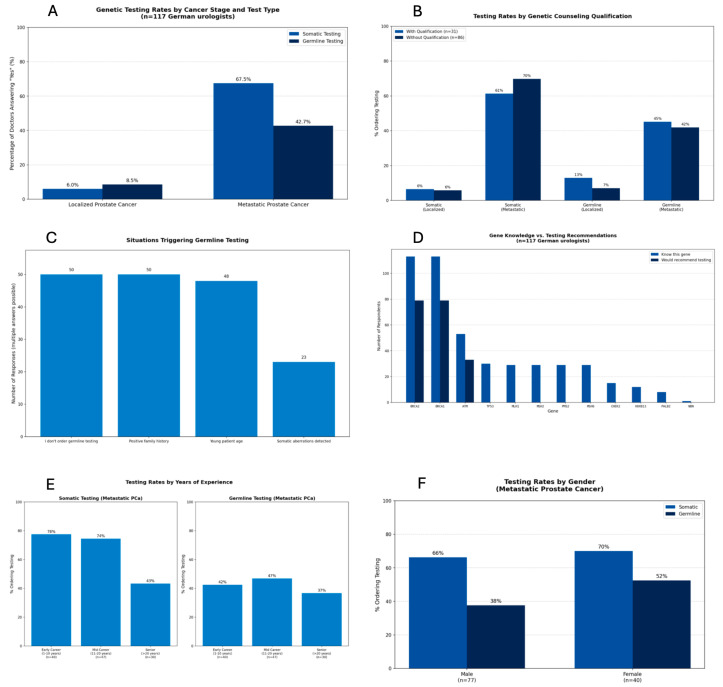
Questionnaire summary. Overview of answers. (**A**) Genetic testing rates by cancer stage and test type: somatic: Q: “Do you order somatic genetic testing for patients with (localized/metastatic) prostate cancer?”, germline: “Do you order germline genetic testing for patients with (localized/metastatic) prostate cancer?” Options: Yes/No. (**B**) Testing rates by genetic counseling qualifications (Grouping variable—Q: “Do you have a qualification in urological cancer-specific genetic counseling?” Testing questions: „Do you order (somatic/germline) genetic testing for (localized/metastatic) prostate cancer?” (**C**) Situations triggering germline testing (Q: “In which situations do you order germline genetic testing for prostate cancer patients?” (Multiple answers possible). (**D**) Gene knowledge vs. testing recommendations: knowledge—Q: “Which genes do you know in connection with hereditary prostate cancer?”; Recommendation: “Which genes would you have tested if recommending germline testing?”(**E**) Testing rates by years of experience: grouping variable—Q: “How long have you been practicing as a specialist?”, testing—Q: “Do you order (somatic/germline) testing for metastatic prostate cancer?”. (**F**) Testing rates by gender: group variable—Q: “Please indicate your gender.”, testing: “Do you order (somatic/germline testing for metastatic prostate cancer?”.

GT was strongly dependent on the disease stage. In localized prostate cancer, seven (6%) GOUs reported performing SoT, and 10 (8.5%) reported performing GeT. For patients with metastatic disease, 79 (67.5%) of the responders reported performing SoT, and 50 (42.7%) reported performing GeT (Figure 2). The difference in testing rates between localized and metastatic disease was statistically significant (*p* < 0.001). Urological cancer-specific genetic counseling qualification had no significant effect on testing behavior. The proportion of GOUs who reported initiating GeT in patients with localized tumors was higher among those with a qualification in urological cancer-specific genetic counseling, though this difference was not statistically significant (12.9% vs. 7.0%, OR 1.98, 95% CI 0.52–7.53, *p* = 0.45; Figure 2A,B).

### 3.3. Clinical Situations Prompting GeT

The clinical situations prompting GOUs to order GeT were strongly concentrated on hereditary risk factors. The most frequent triggers were positive family history (50/117, 42.7%) and young patient age at diagnosis (48/117, 41.0%). Somatic aberrations detected during tumor profiling rarely prompted GeT (23/117, 19.7%). Regarding family history, nearly all respondents asked about prostate cancer (115/117, 98.3%), followed by breast cancer (97/117, 82.9%), ovarian cancer (62/117, 53.0%), colorectal cancer (51/117, 43.6%), and pancreatic cancer (30/117, 25.6%). Notably, 50/117 respondents explicitly stated that they do not order GeT at all (Figure 2C).

### 3.4. Pathways Ordering GeT

The pathways for ordering GeT in clinical routine were most frequently performed through a referral to a human geneticist (46/117, 39.3%). A substantial proportion stated that they do not order GeT themselves (44/117, 37.6%). Direct ordering of GeT by GOU was comparatively uncommon (20/117, 17.1%), while referral to a physician with an additional qualification in urological cancer-specific genetic counseling was reported by 22/117 (18.8%) respondents.

### 3.5. Use of Tumor Boards

Most respondents reported regular use of tumor boards in treatment decision-making (Always: 25/117 (21.4%), Frequently: 61/117 (52.1%), Sometimes: 28/117 (23.9%), Rarely/Never: 3/117 (2.6%) respondents).

### 3.6. Knowledge of Relevant Genetic Alterations

While BRCA1 and BRCA2 were recognized by 96.6% of respondents, knowledge of other prostate cancer-associated genes (ATM 45.3%, CHEK2 12.8%, HOXB13 10.3%, PALB2 6.8%) was much lower. Also, GOUs would only recommend GT for BRCA 1, BRCA 2, and ATM (Figure 2D).

### 3.7. Testing Pattern Differences by Years of Professional Experience and Gender

For SoT in metastatic prostate cancer, early-career and mid-career physicians reported the highest rates (78% and 74%, respectively), whereas senior physicians showed a significantly lower rate (43%; OR 0.46 per career stage, 95% CI 0.27–0.79, *p* = 0.004). For GeT in metastatic disease, rates were higher among mid-career physicians (47%), followed by early-career (42%) and senior physicians (37%), though without statistical significance (OR 0.90 per career stage, 95% CI 0.56–1.46, *p* = 0.67; Figure 2E). Female physicians reported higher rates of GeT and SoT in metastatic disease compared to male physicians (SoT: female 70%, male 66%, OR 0.84, 95% CI 0.37–1.92, *p* = 0.84; GeT: female 52%, male 38%, OR 0.55, 95% CI 0.25–1.18, *p* = 0.17; Figure 2F).

## 4. Discussion

We conducted a nationwide multi-center survey among GOUs to generate real-world evidence on the current state of knowledge and implementation of GT in prostate cancer care in Germany, and to identify potential strategies to improve the uptake of GT and precision oncology.

The respondents’ demographics (age, gender, and professional experience) were well balanced, reasonably representing GOUs in Germany. Fewer female than male GOUs participated (34.2%); however, this proportion is consistent with the gender distribution among urologists in Germany, where 20.6% of all urologists in 2023 were female [10].

We found that nearly one-third of the surveyed GOUs do not perform SoT in patients with metastatic disease (32.5%), and more than half (57.3%) do not perform GeT in their patients. These findings are consistent with those of Castro et al., who collected real-world data from a patient perspective in Europe and found that 37% of mCRPC patients were tested for HRR mutations. While testing rates in Germany were comparatively higher (49%) than in other European countries (e.g., 12% in the U.K.), approximately half of patients remained untested [11]. Correspondingly, Loeb et al. demonstrated a discrepancy between physicians’ knowledge of GeT and actual clinical implementation, as well as limited adherence to national guideline recommendations [12].

Given these guideline recommendations and possible therapeutic consequences (e.g., PARP inhibitors), GT rates should be substantially higher, particularly because prostate cancer patients with HRR/BRCA mutations have an inferior prognosis on standard therapy alone. In a real-world German cohort, Wenzel et al. reported a significantly worse overall survival for HRRm-positive and BRCA 1/2-positive patients in metastatic hormone-sensitive prostate cancer and mCRPC [13]. Crucially, the use of precision oncology therapies, such as PARP inhibitors, can significantly improve the overall survival of these high-risk patients. In the PROfound trial, olaparib significantly extended overall survival in BRCA1/2-positive and ATM-mutated mCRPC patients [14]. Similarly, adding talazoparib to enzalutamide extended overall survival in HRRm-positive mCRPC patients [15]. Only recently, the benefit of PARP inhibitor use in earlier therapeutic sequences was found, combining niraparib/abiraterone in metastatic hormone-sensitive prostate cancer for BRCA 1/2-positive patients, which significantly improved radiologic progression-free survival [16]. Without GT, patients with genetic aberrations remain undetected and are treated with standard therapies to which their tumors are less responsive.

In our study, only 8.5% of the surveyed GOUs would perform GeT in localized disease, despite more GOUs being aware of relevant risk factors that would justify GeT in this setting (Figure 2C). This may have negative implications for both the individual patient and the identification of family members at increased hereditary cancer risk, potentially limiting access to genetic counseling and preventive strategies. Regardless of disease stage, 37.6% of GOUs neither refer patients for it nor perform GeT at all. However, this finding needs to be interpreted in the context of the German S3 guideline. At the time the survey was conducted, the German S3 guideline did not yet include specific recommendations on GeT, as these were only introduced with the 2025 update, right after completing the survey. The low uptake of GeT in localized disease may therefore reflect the absence of clear national guidance as well as possible concerns about heterogeneous insurance coverage. Concurrently, this pattern suggests that GOUs primarily align clinical practice with the S3 guideline, rather than incorporating broader recommendations from international guidelines. The additional qualification in urological cancer-specific genetic counseling represents an attempt to facilitate GT outside human genetics departments. However, we found no statistically significant difference in GT utilization between GOUs with and without this qualification. As the study was not powered to detect such differences, this finding should be interpreted carefully.

Moreover, we observed a significant negative association between years of professional experience and the proportion of GOUs performing GT. Early- and mid-career physicians reported higher rates of GT in their patients than senior colleagues. This generational disparity likely represents differences in training exposure, as precision oncology and GT have only recently been integrated as important components of genitourinary cancer care. Moreover, limited familiarity with international guidelines published in English may also represent a potential barrier. As these factors were not assessed, the underlying reasons remain uncertain and require further investigation. There was a non-significant trend toward more female GOUs performing GT compared to their male colleagues. Notably, other studies from the U.S. and Switzerland found that female physicians demonstrate higher adherence to guideline-recommended GT and tend to deliver superior preventive care [17,18,19].

Certain limitations of our study should be acknowledged. The questionnaire did not assess how GOUs became aware of the survey. Therefore, the effectiveness of the different distribution channels and specific response rates could not be assessed. Moreover, the characteristics of non-responders remain unknown. The survey is based on self-reported data of GOUs and may therefore be subject to over- or underestimation, while it remains uncertain to what extent reported practice behaviors are consistently applied in routine outpatient care. Subjective surveys of physicians cannot provide a complete picture of real-world patient care. In addition, selection bias cannot be excluded, as participating urologists are likely to have a greater interest or awareness of GT in prostate cancer. The sample size of our study is relatively small in relation to the total number of practicing urologists in Germany. However, it is comparable to other international studies on this topic. Loeb et al. received 132 responses from urologists across the United States in their survey on knowledge and practice of GeT in prostate cancer, suggesting that our sample size is approximately in line with the existing literature [12]. Our survey did not systematically assess the specific barriers underlying the limited use of GT. Future surveys should evaluate logistical, professional, structural, and patient-related barriers in greater detail to enable the development of targeted improvement strategies.

All in all, our data demonstrate a clinically relevant gap between guideline recommendations and real-world practice in Germany. Showing this gap is an important first step toward the development of educational initiatives, guideline-based decision diagrams, standardized patient information, and structured referral pathways for genetic counseling. Strengthening interdisciplinary collaboration between outpatient urologists, uro-oncological centers, and human genetics services may further promote the implementation of GT in routine care.

Notably, experience from other tumor entities, such as BRCA-associated breast and ovarian cancer, has demonstrated that the integration of GT and counseling at early stages of disease is feasible within routine clinical practice. Improving testing rates in other tumor entities relied on the following principles: universal testing recommendations in the guidelines [20], genetic counselors in cancer care departments [21], digital solutions for specific genetic risk assessment [22], and education of physicians and patients [23,24]. Similarly, there are already different approaches to address this global issue in prostate cancer care, such as telehealth [25], video education of clinicians [26] and patients [27], and guidelines-based digital tools to assess the need for GeT based on risk factors [28]. In some countries, implementation efforts focused on mainstreaming models, where managing clinicians without additional qualification are trained to initiate GT while the interpretation is performed with a specialist [29,30,31].

## 5. Conclusions

In conclusion, despite clear international guideline recommendations, the implementation of GT, particularly GeT, in prostate cancer care remains inconsistent. Our findings highlight the need for more structured clinical frameworks, educational initiatives, and streamlined diagnostic pathways to support GOUs and patients to accelerate the implementation of GT and precision medicine into routine prostate cancer care.

## Figures and Tables

**Table 1:** Survey on genetic testing in patients with prostate cancer (Abbreviations: HRR = homologous recombination repair, MMR = mismatch repair, DNA = deoxyribonucleic acid, CRC = colorectal cancer, Ca = carcinoma, * = multiple answers possible).

Question		Response Options
**Q1**	Sex	Male; Female
**Q2**	Age	<44; 45–54; 55–64; >65
**Q3**	Years of experience as a board-certified urologist in Germany	1–5; 6–10; 11–15; 16–20; 21–25; 26–30; >30 years
**Q4**	Workplace	Private practice; Outpatient center; Hospital
**Q5**	Federal state	Baden-Wuerttemberg, Bavaria, Berlin, Brandenburg, Bremen, Hamburg, Hesse, Mecklenburg-Western Pomerania, Lower Saxony, North Rhine-Westphalia, Rhineland-Palatinate, Saarland, Saxony, Saxony-Anhalt, Schleswig-Holstein, Thuringia
**Q6**	Specialist training	Urology; Medical oncology; Andrology; Other
**Q7**	Qualification in Urological Cancer-Specific Genetic Counseling	Yes; No
**Q8**	Frequency of treating metastatic PCa	Daily; Weekly; Monthly; Rarely
**Q9**	Somatic testing in localized PCa	Yes; No
**Q10**	Somatic testing in metastatic PCa	Yes; No
**Q11**	Germline testing in localized PCa	Yes; No
**Q12**	Germline testing in metastatic PCa	Yes; No
Q13 *	Indication for germline testing	Somatic aberrations; Positive family history; Young age; No germline testing
Q14 *	Pathways ordering of germline testing	Referral to medical genetics; Referral to qualified genetic counselor; Direct lab order; None
Q15 *	Recommended genes/panels for germline testing	BRCA1;2; BRCA1;2 + ATM; PCa-specific panels; HRR panel; HRR + MMR panel; None; Unsure
Q16 *	Known genes in hereditary PCa	BRCA1; BRCA2; ATM; CHEK2; TP53; PALB2; HOXB13; MMR genes; NBN; None; Unsure
Q17 *	Family history	ProstateCa; Breast Ca; Ovarian Ca; CRC; Urothelial Ca; Pancreatic Ca; Melanoma; Other; No inquiry
**Q18**	Use of tumor boards	Always; Often; Sometimes; Rarely; Never

## Data Availability

All data generated or analyzed during this study are included in this article and its Supplementary Material Files. Further inquiries can be directed to the corresponding author.

## Supplementary Materials

The following supporting information can be downloaded at: https://www.mdpi.com/article/10.3390/cancers18132030/s1, Table S1. Gene abbreviations and full names of the genes.

## Abbreviations

The following abbreviations are used in this manuscript:

GT	Genetic testing
PC	Prostate cancer
GOU	German Outpatient Urologists
SoT	Somatic testing
GeT	Germline testing
HRR	Homologous recombination repair
MMR	Mismatch repair
mCRPC	Metastatic castration resistant prostate cancer
