# Supplementary material for: Implementation of Genetic Testing in Prostate Cancer: A Real-World Survey of Outpatient Urologists in Germany (PRO-GEN)
Source: Cancers (Basel). 2026 Jun 23;18(13):2030. doi: 10.3390/cancers18132030 (PMC13359895; doi:10.3390/cancers18132030)
Supplement: Supplementary file 1 [file cancers-18-02030-s001.zip › cancers-4364090-supplementary.pdf]

Supplementary Table S1. Gene abbreviations and full names of the genes.

Gene	Official full gene name
BRCA1	Breast Cancer 1
BRCA2	Breast Cancer 2
ATM	ATM serine/threonine kinase
TP53	tumor protein p53
MLH1	mutL homolog 1
MSH2	mutS homolog 2
PMS2	PMS1 homolog 2, mismatch repair system component
MSH6	mutS homolog 6
CHEK2	checkpoint kinase 2
HOXB13	homeobox B13
PALB2	partner and localizer of BRCA2
NBN	nibrin
